# Immunotherapy Alone or in Combination with Stereotactic Body Radiotherapy in Advanced Lung Cancer: A Pooled Analysis of Randomized Clinical Trials

**DOI:** 10.1155/2022/7506300

**Published:** 2022-09-20

**Authors:** Bi-Cheng Wang, Bo-Hua Kuang, Guo-He Lin

**Affiliations:** ^1^Cancer Center, Union Hospital, Tongji Medical College, Huazhong University of Science and Technology, Wuhan 430022, China; ^2^Department of Oncology, The Second Affiliated Hospital of Anhui Medical University, Hefei 230601, China

## Abstract

**Background:**

Immunotherapy has revolutionized the treatment of advanced lung cancer. Nevertheless, it remains unclear whether adding stereotactic body radiotherapy (SBRT) to immunotherapy (IT) further improves responses and survival outcomes. Therefore, in this pooled analysis, we comprehensively compared IT plus SBRT with IT alone in patients with advanced lung cancer.

**Methods:**

Online databases, including PubMed, Web of Science, Embase, and Cochrane CENTRAL, were systematically searched on April 24, 2022. Eligible studies were randomized clinical trials comparing IT plus SBRT to IT. The primary outcomes were the objective response rate (ORR) and disease control rate (DCR). Progression-free survival (PFS) and overall survival (OS) were explored as secondary outcomes.

**Results:**

Overall, three phase 2 randomized clinical trials with a total of 146 previously treated lung cancer patients were enrolled. The median PFS and OS were 3.8 months and 9.5 months for IT plus SBRT versus 2.4 months and 6.1 months for IT. Comparing IT plus SBRT with IT alone, pooled risk ratios for ORR and DCR were 1.95 (95% confidence interval 1.07–3.53, *p* = 0.03) and 1.28 (0.94–1.73, *p* = 0.12). While pooled hazard ratios were 0.77 (0.25–2.42, *p* = 0.66) for PFS and 0.71 (0.16–3.21, *p* = 0.65) for OS, respectively. No publication bias was found across the trials.

**Conclusion:**

Compared to IT alone, the addition of SBRT improved the best response but failed to prolong the survival outcomes in treating advanced lung cancer patients. Future studies are necessary to explore new modalities of the combination of IT and SBRT.

## 1. Introduction

Immune checkpoint inhibitors, including antiprogrammed cell death 1 (PD-1), programmed cell death-ligand 1 (PD-L1), and cytotoxic T lymphocyte-associated protein 4 (CTLA-4) agents, have been widely applied in patients with non-small-cell lung cancer (NSCLC) and small cell lung cancer (SCLC). Nevertheless, most lung cancer patients benefit limitedly from mono-immunotherapy (IT). Therefore, investigating IT-based combination treatments is necessary to elevate the efficacy and prolong the survival outcomes.

For relapsed or metastatic lung cancer, combining IT with stereotactic body radiotherapy (SBRT) has been hoped. Three to five fractions of high-dose radiation therapy (≥5 Gy/fraction) may increase tumor antigen release and antigen presentation and improve T-cell infiltration in irradiated lesions (1-4). Theoretically, the addition of SBRT could enhance the antitumor effects of IT.

In a phase 1 trial reported by Bestvina, IT combined with SBRT showed a 46% objective response rate (ORR) with a median progression-free survival (PFS) of 5.8 months in widely metastatic NSCLC patients (5). In another phase 1 trial reported by Ye, the ORR was 39% and the median PFS was six months when advanced lung cancer patients were treated with IT plus SBRT (6). Moreover, several case studies indicated that some advanced patients achieved long-term survival after IT and SBRT (7, 8).

However, the multicenter, randomized, phase 2 trial published by Schoenfeld compared IT alone with IT plus SBRT, and did not find a significant improvement of adding SBRT to IT on responses in advanced NSCLC patients (ORR: 11.5% in the IT plus SBRT group versus 11.5% in the IT group) (9). Accordingly, these results remind clinicians to rethink the efficacy of combining IT and SBRT.

Thus, we conducted a pooled analysis to comprehensively evaluate the combination of IT and SBRT versus IT alone in advanced lung cancer patients.

## 2. Methods

This analysis was conducted according to the Preferred Reporting Items for Systematic Reviews and Meta-analyses (PRISMA) guidelines (10).

### 2.1. Search Strategy

A systematic literature search was performed in online databases, including PubMed, Web of Science, Embase, and Cochrane CENTRAL, on April 24, 2022. Search terms were (lung cancer) AND (stereotactic OR hypofractionated) AND (radiotherapy OR radiation OR radiosurgery) AND (immunotherapy OR immune checkpoint OR PD-1 OR PD-L1 OR CTLA-4). References to relevant records in references were reviewed for more eligible trials.

### 2.2. Selection Criteria

All of the eligible clinical trials should meet the following inclusion criteria: (1) Patients had histological or cytological confirmed advanced lung cancer patients (11, 12), (2) Patients were treated with IT plus SBRT versus IT alone, and ≥5 Gy for each fraction was mandatory, (3) prospective and randomized studies, (4) enrolled studies were published in English.

Exclusion criteria were (1) single-arm studies, (2) reviews/comments/letters, (3) meeting abstracts, (4) IT plus SBRT versus SBRT studies, (5) study protocols, (6) case reports, and (7) retrospective studies. Any disagreements were resolved by discussion.

### 2.3. Data Extraction and Quality Assessment

The primary outcomes were ORR and disease control rate (DCR), and the secondary outcomes were overall survival (OS) and PFS. Two of us (Bi-Cheng Wang and Bo-Hua Kuang) independently extracted detailed data from the eligible clinical trials, comprising first author, year of publication, study design, tumor type, previous line of therapy, number of patients, therapeutic strategies, responses, survival outcomes, and toxicities. The Engauge Digitizer software and the statistic formula reported by Jayne F Tierney were applied to reconstruct the time-to-event data that were not directly reported in the original articles (13). The latent publication bias among the studies was evaluated through Egger's tests.

### 2.4. Statistical Analysis

ORR and DCR data were evaluated by risk ratio (RR) with 95% confidence intervals (CIs). While data of OS and PFS were assessed by hazard ratio (HR) with 95% Cis, respectively. R software (version 4.1) and the “meta” package was adopted to synthesize the responses and survivals.

Median survival data were pooled-analyzed by STATA software (version 14.0) and “metan” code. To calculate the not reached up IC data, “up IC = median + (median–low IC)” formula was adopted.

Heterogeneities were assessed by t^2^ and *I*^2^ statistic percentages. Both fixed-effect and random-effects models were used. However, when heterogeneity was low (*I*^2^ < 50% or *p* value < 0.1), the pooled analysis was applied through a fixed-effect model with the Mantel–Haenszel method. Otherwise, a random-effects model was selected. Differences with *p* values < 0.05 for ORR, DCR, OS, and PFS were considered statistically significant.

## 3. Results

### 3.1. Eligible Clinical Trials and Basic Characteristics

**Figure 1:**
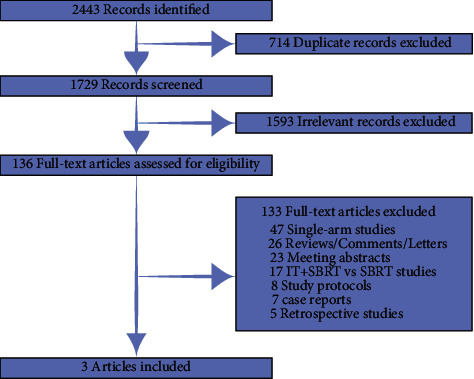
Process of selecting eligible clinical trials.

Our search of the online databases (PubMed, Web of Science, Embase, and Cochrane CENTRAL) identified 2443 relevant records. 714 duplicated records were excluded. 1593 irrelevant records were excluded after screening the titles and abstracts. 136 full-text articles were assessed for eligibility. Subsequently, 47 single-arm studies, 26 reviews/comments/letters, 23 meeting abstracts, 17 IT + SBRT vs. SBRT studies, 8 study protocols, 7 case reports, and 5 retrospective studies were excluded. Finally, three phase 2 randomized clinical trials with 146 advanced lung cancer patients were reviewed and pooled-analyzed (Figure 1) (9, 14, 15).

Table 1 depicts the characteristics of the eligible clinical trials. All enrolled patients had received at least one line of previous systemic therapy. IT strategies included pembrolizumab (200 mg/kg, q3w) in Theelen's trial and durvalumab (1500 mg, q4w) plus tremelimumab (75 mg, q4w) in Pakkala's and Schoenfeld's trials. In terms of SBRT, 24 Gy/3 Fractions and 27 Gy/3 Fractions were administered.

### 3.2. Responses

**Figure 2:**
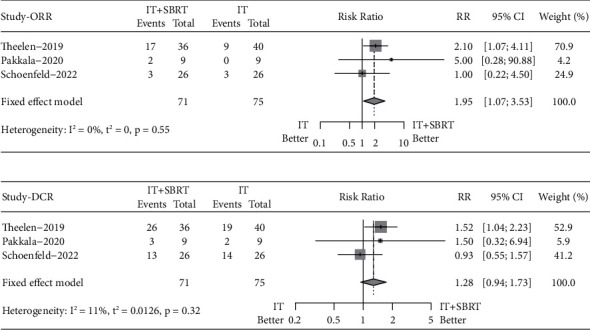
Forest plots of the pooled risk ratios for objective response rate (a) and disease control rate (b) between immunotherapy plus stereotactic body radiotherapy and immunotherapy alone.

The pooled RR for ORR was 1.95 (95% CI 1.07–3.53, Fixed-effect model, *p* = 0.03), indicating that adding SBRT to IT significantly improved the best response rate compared to IT alone (Figure 2(a)).

In terms of DCR, the pooled RR was 1.28 (95% CI 0.94–1.73, Fixed-effect model, *p* = 0.12), demonstrating that both strategies had comparable DCRs (Figure 2(b)).

### 3.3. Survival Outcomes

Table 2 depicts the survival outcomes. The median PFS for IT plus SBRT was 3.8 months (95% CI 2.3–5.3) versus 2.4 months (95% CI 1.4–3.3) for IT alone. The median OS was 9.5 months (95% CI 6.1–13.0) in the IT plus SBRT group and 6.1 months (95% CI 2.8–9.3) in the IT group.

HR and 95% CI data in Theelen's and Schoenfeld's clinical trials could be extracted directly from the original articles. While the time-to-event data from Pakkala's trial were reproduced according to the PFS and OS curves. Comparing IT plus SBRT versus IT alone, the reproduced HR for PFS was 0.71 (95% CI 0.34–1.48) and for OS was 1.39 (95% CI 0.14–13.39).

**Figure 3:**
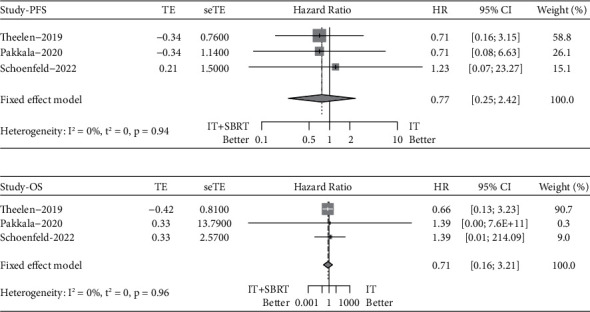
Forest plots of the pooled hazard ratio for progression-free survival (a) and overall survival (b) between immunotherapy plus stereotactic body radiotherapy and immunotherapy alone.

The pooled HR for PFS was 0.77 (95% CI 0.25–2.42, Fixed-effect model, *p* = 0.66), illustrating that IT plus SBRT failed to significantly prolong PFS compared with IT alone (Figure 3(a)).

The pooled HR for OS was 0.71 (95% CI 0.16–3.21, Fixed-effect model, *p* = 0.65). The forest plot showed that advanced lung cancer patients obtained similar OS benefits from IT plus SBRT versus IT alone (Figure 3(b)).

### 3.4. Risk of Publication Bias

**Figure 4:**
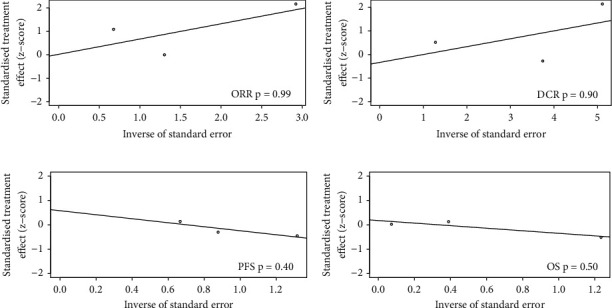
Latent publication bias among the eligible clinical trials.

Figure 4 displayed the latent publication bias through Egger's tests in the pooled analyses of ORR, DCR, PFS, and OS, and no bias across the trials was reported.

## 4. Discussion

In this pooled analysis of randomized clinical trials, the combination of IT and SBRT improved the ORR (RR 1.95, 95% CI 1.07–3.53, *p* = 0.03) but did not significantly prolong the PFS (3.8 months versus 2.4 months, HR 0.77, 95% CI 0.25–2.42, *p* = 0.66) and OS (9.5 months versus 6.1 months, HR 0.71, 95% CI 0.16–3.21, *p* = 0.65) against IT alone in advanced lung cancer. These results could provide useful information for future studies.

Similar negative results have been found in head and neck squamous cell carcinoma (HNSCC). In McBride's trial, 62 metastatic HNSCC patients were randomized to receive nivolumab (3 mg/kg, q2w) plus SBRT (27 Gy/3 Fractions) or nivolumab alone. The data showed no significant improvements between the groups, and no abscopal effects were observed with the addition of SBRT to IT (16). Herein, the combination of IT and SBRT may be facing great challenges.

Two reasons can explain the challenges. All the enrolled patients in our analysis had received at least one previous systemic therapy, and more than two metastatic lesions existed. The background of these patients may indicate the low responses to IT or SBRT. On the other hand, we deduced that the main systemic effects might be produced by IT, and that SBRT could be effective only for the target site.

A reasonable time and manner for adding SBRT are essential. In the first-line setting, systemic therapy (chemotherapy, immunotherapy, or targeted therapy) followed by SBRT for primary lesions showed an 82% ORR and a 100% DCR with a median PFS of 14 months (17). Thus, SBRT for residual primary lesions after first-line systemic therapy for advanced NSCLC could have satisfied responses.

In oligoprogressive advanced disease (NSCLC and melanoma), IT (nivolumab) combined with SBRT had a 42% ORR with median PFS and OS of 14.2 and 37.4 months (18). Accordingly, SBRT to oligoprogressive lesions can improve local control and delay further disease progression in advanced lung cancer patients (19).

For patients with early-stage disease, the combination of IT and SBRT could be a novel neoadjuvant strategy. In Altorki's randomized phase 2 trial, early-stage NSCLC patients received either IT (durvalumab) plus SBRT (24 Gy/3 Fractions) or IT (durvalumab) alone. The results showed that IT plus SBRT was associated with a critically higher response rate than IT alone (53% versus 7%) (20).

In addition, granulocyte-macrophage colony stimulating factor (GM-CSF) may be a potential factor in enhancing the efficacy of IT plus SBRT (21, 22). In Ni's report, patients with advanced NSCLC who had failed first-line systemic therapy were treated with IT (sintilimab 200 mg, q3w) plus SBRT (24 Gy/3 Fractions) plus GM-CSF (125 *μ*g/m^2^d1-14, q3w). With a median follow-up of 7.9 months, the ORR was 35%, and the median PFS was 6.9 months (23). Even though the addition of GM-CSF showed promising efficacy in the second-line treatment of NSCLC, randomized clinical trials are needed to validate this novel therapeutic modality.

Although SBRT failed to prolong the PFS and OS based on IT, SBRT is undoubtedly an effective treatment for local disease control. For patients with brain metastasis, SBRT of brain lesions reduced the incidence of leptomeningeal seeding (from 93.2% to 69.1%) (24), indicating that brain metastasis patients might benefit from the addition of SBRT to IT (25).

### 4.1. Limitations

There were several limitations to this analysis. (1) Sample size was small since only 146 patients were enrolled. However, all eligible studies were well-designed randomized phase 2 clinical trials, and no heterogeneities and publication bias were found among the studies. Thus, we believe that our pooled results may be enough to demonstrate the comparable effects between IT plus SBRT and IT alone and could provide helpful and valuable information to clinicians in their future clinical practice. (2) The backgrounds of the participants were different, including previous treatments, sites of metastatic tumors, and performance status. Nevertheless, IT combined with SBRT could be a suitable option for certain patients. (3) Safety data were insufficient for pooled analysis, and no severe toxic effects had been reported after the addition of SBRT.

## 5. Conclusion

In this study, we pooled-analyzed published data to compare IT plus SBRT with IT and found that the best responses were significantly improved. In addition, the combination therapy showed longer median PFS and OS versus monotherapy, even though the differences were not statistically significant at the current stage. Accordingly, our results may provide evidence of an added benefit with the addition of SBRT to IT in advanced lung cancer patients. Through our study, we intend to emphasize the feasibility of the IT plus SBRT combination strategy and to encourage clinicians to detect more effective IT/SBRT-related therapeutic modalities (including optimal radiotherapy dose or timing, and immune checkpoint agents.) in suitable patient cohorts.

## Figures and Tables

**Table 1:** Characteristics of enrolled clinical trials.

First author	Year of publication	Design	Tumor type	Previous line of therapy	Number of patients	IT	SBRT
Theelen	2019	A phase 2 randomized clinical trial	NSCLC	1 : 75%	IT + SBRT: 40	Pembrolizumab: 200 mg/kg, q3w,	24 Gy/3 fractions
			≥2 : 25%	IT: 36			

Pakkala	2020	A phase 2 randomized clinical trial	SCLC	1 : 72%	IT + SBRT: 9	Durvalumab: 1500 mg, q4w, for 12 months	27 Gy/3 fractions
			IT: 9	≥2 : 28%		Tremelimumab: 75 mg, q4w, for up to 4 cycles	

Schoenfeld	2022	A phase 2 randomized clinical trial	NSCLC	1 : 0%	IT + SBRT: 26	Durvalumab: 1500 mg, q4w, for up to 13 cycles	24 Gy/3 fractions
				≥2 : 100%	IT: 26	Tremelimumab: 75 mg, q4w, for up to 4 cycles	

Abbreviations. IT, immunotherapy; SBRT, stereotactic body radiotherapy.

**Table 2:** Survival outcomes in eligible studies.

Study	Groups	Median OS	Median PFS
Theelen-2019	IT + SBRT IT	15.9 months (95% CI 7.1-not reached) 7.6 months (95% CI 6.0–13.9)	6.6 months (95% CI 4.0–14.6) 1.9 months (95% CI 1.7–6.9)

Pakkala-2020	IT + SBRT IT	5.7 months (95% CI 1.6–14.5) 2.8 months (95% CI 0.8–12.4)	3.3 months (95% CI 0.9–4.9)2.1 months (95% CI 0.8–3.2)

Schoenfeld-2022	IT + SBRTIT	9.7 months (95% CI 5.1-not reached) Not reached (95% CI 4.9-not reached)	4.0 months (95% CI 2.1–7.0) 3.3 months (95% CI 1.8–5.5)

Pooled survivals	IT + SBRT IT	9.5 months (95% CI 6.1–13.0) 6.1 months (95% CI 2.8–9.3)	3.8 months (95% CI 2.3–5.3) 2.4 months (95% CI 1.4–3.3)

Abbreviations. IT, immunotherapy; SBRT, stereotactic body radiotherapy; OS, overall survival; PFS, progression-free survival.

## Data Availability

All eligible clinical trials can be searched and downloaded from their official websites. (1) 10.1016/s1470-2045(21)00658–6 (2). 10.1136/jitc-2020-001302 (3) 10.1001/jamaoncol.2019.1478.
